# Sleep and schizophrenia polygenic scores in non-affective and affective psychotic disorders

**DOI:** 10.1017/S0033291725000844

**Published:** 2025-04-15

**Authors:** Erik Cederlöf, Minna Holm, Anders Kämpe, Ari Ahola-Olli, Katri Kantojärvi, Markku Lähteenvuo, Johan Ahti, Jarmo Hietala, Katja Häkkinen, Erkki Isometsä, Annamari Tuulio-Henriksson, Olli Kampman, Kaisla Lahdensuo, Jouko Lönnqvist, Jari Tiihonen, Hannu Turunen, Asko Wegelius, Juha Veijola, Tuula Kieseppä, Aarno Palotie, Tiina Paunio

**Affiliations:** 1 Finnish Institute for Health and Welfare, Helsinki, Finland; 2 Department of Psychiatry, University of Helsinki and Helsinki University Hospital, Helsinki, Finland; 3 SleepWell Research Program, Faculty of Medicine, University of Helsinki and Helsinki University Hospital, Helsinki, Finland; 4 Institute for Molecular Medicine FIMM, University of Helsinki, Helsinki, Finland; 5 Niuvanniemi Hospital, University of Eastern, Kuopio, Finland; 6 Faculty of Medicine, University of Turku, Turku, Finland; 7 Department of Psychology and Logopedics, Faculty of Medicine, University of Helsinki, Helsinki, Finland; 8 Department of Psychiatry, The Pirkanmaa Wellbeing Services County, Tampere, Finland; 9 Faculty of Medicine and Health Technology, University of Tampere, Tampere, Finland; 10 Department of Clinical Sciences, Psychiatry, Umeå University, Umeå, Sweden; 11 Department of Psychiatry, The Wellbeing Services County of Ostrobothnia, Vaasa, Finland; 12 Department of Clinical Neuroscience, Karolinska Institutet, Stockholm, Sweden; 13 Department of Psychiatry, Research Unit of Clinical Neuroscience, University of Oulu, Oulu, Finland; 14 Medical Research Center, University of Oulu and Oulu University Hospital, Oulu, Finland; 15 Finnish Ministry of Social Affairs and Health, Helsinki, Finland

**Keywords:** cognition, polygenic scores, psychotic disorders, schizophrenia, sleep

## Abstract

**Background:**

Sleep problems are common in psychotic disorders and are associated with worse quality of life and disease prognosis. Genome-wide association studies (GWAS) have revealed genetic influences for schizophrenia and sleep, but polygenic scores (PGSs) for sleep traits have not been evaluated systematically in patients with psychotic disorders.

**Methods:**

This study investigated the associations between PGSs for sleep traits (insomnia, PGS_INS_; sleep duration, PGS_SD_; short sleep duration, PGS_SS_; long sleep duration; PGS_LS_), diurnal preference (eveningness, PGS_ME_), and schizophrenia (PGS_SZ_) with clinical features of psychotic disorders in the Finnish SUPER study comprising 8,232 patients with psychotic disorders. The measures included self-reported sleep and well-being, cognitive assessments, clozapine use, and functional outcomes. Using FinnGen data of 356,077 individuals, we analyzed the distributions of PGSs in psychotic and bipolar disorders and the general population.

**Results:**

PGS_INS_ associated with more sleep problems and worse well-being (e.g. worse health-related quality of life [β = −0.07, CI = −0.09, −0.05, *p* < .001]). High PGS_SZ_ is associated with better sleep quality, worse clinical outcomes, and performance in cognitive tests (e.g. more errors in paired-associated learning [β = 0.07, CI = 0.04, 0.09, *p* < .001]). PGS_INS_ was higher in affective psychotic and bipolar disorders, while PGS_SD_ and PGS_ME_ were higher in schizophrenia as compared with individuals with no psychiatric disorders.

**Conclusion:**

Genetic risks for sleep and diurnal preference vary between non-affective psychosis, affective psychosis, and the general population. The findings in this study emphasize the heterogeneity in genetic etiology of the objective features of disease severity and the more subjective measures related to well-being and self-reported measures of sleep.

## Introduction

Schizophrenia is among the most severe and debilitating psychiatric disorders, significantly impacting the quality of life and general functioning (Dong et al., 2019; Green, Kern, & Heaton, 2004; Holm, Taipale, Tanskanen, Tiihonen, & Mitterdorfer-Rutz, 2021; Saarni et al., 2010). It is characterized by a combination of neuropsychological mechanisms that result in disturbed mental processes (Palmer, Dawes, & Heaton, 2009). Cognitive impairment is one of the most robust endophenotypes in schizophrenia and is also common in patients with other psychotic disorders (including schizoaffective disorder, bipolar disorder, and psychotic depression) (Barch & Ceaser, 2012; Green & Harvey, 2014; Lepage, Bodnar, & Bowie, 2014; Li et al., 2020).

Patients with psychotic disorders often experience sleep problems, including insomnia and hypersomnia symptoms (Freeman, Sheaves, Waite, Harvey, & Harrison, 2020; Krystal, 2012; Reeve, Sheaves, & Freeman, 2019, 2021). In our previous study, patients with affective psychotic disorders exhibited more insomnia symptoms than patients with schizophrenia, who more often experienced excessively long sleep (37% of patients with schizophrenia, compared with 24% of patients with psychotic depression, as derived from previously published data). These sleep problems are associated strongly with worse subjective health (Cederlöf et al., 2022). Both sleep problems and cognitive impairments have been linked to negative outcomes, including a worse quality of life (DeRosse, Nitzburg, Blair, & Malhotra, 2018; Ritsner, Kurs, Ponizovsky, & Hadjez, 2004).

Genome-wide association studies (GWAS) have identified multiple risk variants for schizophrenia (Trubetskoy et al., 2022) and other psychiatric disorders and traits, leading to the development of polygenic scores (PGSs). PGS for schizophrenia (PGS_SZ_) has a considerable explanatory effect for schizophrenia and weaker but significant associations also with schizoaffective disorder and bipolar disorder type 1 (Allardyce et al., 2018; Sullivan et al., 2018). A high PGS_SZ_ has in patients with schizophrenia been previously associated with poorer quality of life (Pazoki et al., 2020), more involuntary hospitalizations (Meier et al., 2016), and clozapine use (Lin et al., 2023). Poor cognitive functioning has been associated with PGS_SZ_ in the general population and first-episode psychosis cohorts, but not in schizophrenia(Jonas et al., 2019; Mallet, Strat, Dubertret, & Gorwood, 2020; Richards et al., 2020). Studies on associations between PGS_SZ_ and sleep in adulthood are scarce. One study with a general population sample, including a subsample of patients with schizophrenia, found associations between PGS_SZ_ and early morning awakenings, reduced sleep efficiency, and increased napping (Wainberg et al., 2021).

Well-established PGSs for sleep traits include those for insomnia (PGS_INS_) (Lane et al., 2017) and sleep duration (PGS_SD_) (Dashti, Redline, & Saxena, 2019), and for diurnal preference (PGS_ME_) (Jones et al., 2019; Wray et al., 2014; Zhang, Privé, Vilhjálmsson, & Speed, 2021). PGSs for short (PGS_SS_) and long sleep duration (PGS_LS_) have also recently been created (Austin-Zimmerman et al., 2023). These PGSs have been derived from self-report data in the general population, in whom they have been shown to have a relatively modest impact on sleep-related measures. Sleep PGSs have in genetic correlation studies been found to correlate with various psychiatric disorders and traits, including both affective disorders and PGS_SZ._ (; Dashti, Redline, & Saxena, 2019; Jansen et al., 2019; Jones et al., 2019; O’Connell et al., 2021). Few studies on sleep or diurnal preference PGSs in psychiatric disorders have been done. In one study, high PGS_SD_ was not associated with schizophrenia or bipolar disorder (Dashti et al., 2019).

In this study, we aimed to compare genetic influences on objective measures of disease severity and subjective measures related to life quality and sleep in patients with psychotic disorders in a systematically collected nationwide sample. Specifically, we aimed to clarify the relationship between PGSs of sleep, diurnal traits, and schizophrenia, with (1) self-reported measures on sleep, subjective health, and cognitive functioning and (2) objective measures including involuntary hospitalizations and cognitive performance) in a large sample of patients with psychotic disorders. We hypothesized that genetic predisposition for sleep and diurnal traits are primarily related to subjective measures, while that for schizophrenia is mainly related to objective measures. Finally, to potentially shed light on the mechanisms of sleep problems in psychotic disorders, we (3) explored differences in these PGSs in non-affective and affective psychotic disorders, as compared with individuals with no psychiatric disorders from the general population.

## Materials and methods

### Study sample

This study is part of the SUPER research project, which examines psychotic disorders. The SUPER project is part of the international Stanley Global Neuropsychiatric Genomics Initiative. In Finland, the Institute for Molecular Medicine Finland (FIMM), the Finnish Institute of Health and Welfare (THL), and the University of Helsinki oversaw the research project. The project was carried out in cooperation with all hospital districts in Finland.

### SUPER cohort

Adult Finnish patients with schizophrenia spectrum disorders (ICD-10 codes: F20–F29), bipolar disorder, or psychotic depression (ICD-10 codes: F30.2, F31, F32.3, and F33.3) with a history of at least one psychotic episode were eligible to participate. The diagnoses were retrieved from the Finnish Care Register for Health Care (HILMO). Finnish registry data have been shown to be of high-quality, including for mental health diagnoses (Sund, 2012). In the present study, patients with (1) schizophrenia, (2) schizoaffective disorder, (3) bipolar disorder, or (4) psychotic depression were included. These diagnoses were categorized into diagnostic groups of (1) schizophrenia and (2) affective psychotic disorders, including schizoaffective disorder, bipolar disorder, and psychotic depression. The questionnaire, interview, cognitive tests, and genetic sampling were all conducted during the same session with a study nurse. The complete study protocol and further information about the study population were published previously (Ahola-Olli et al., 2023).

The total sample size was 10,411 patients. Patients with an unknown or other diagnosis than the four diagnostic groups, those who did not complete the questionnaire, were older than 80 years (or no age registered), or had no or subpar genotypic information, were excluded. After exclusion 8,232 patients remained. For the study sample flow chart, see Supplementary Figure 1.

### PGS calculation

All PGSs were based on PRS-CS (Ge, Chen, Ni, Feng, & Smoller, 2019), using the largest publicly available summary statistics for each trait. These included the PGS for insomnia (Lane et al., 2017), sleep duration (Dashti, Redline, & Saxena, 2019), schizophrenia (Trubetskoy et al., 2022), as well as the PGSs for long and short sleep duration (Austin-Zimmerman et al., 2023). The PGS for eveningness was derived from a PGS originally developed for morningness (Jones et al., 2019). The entire genome was used, meaning all reliably imputed SNPs that intersected with each trait’s GWAS summary statistics were included. The Illumina Global Screening Array was used to genotype all participants. Samples where the inferred sex was mismatched with the recorded sex were excluded, as were samples with poor genotype quality. Imputation was performed using a Finnish-specific reference panel SISu version 3 (Pärn et al., n.d.) and only reliably imputed variants (INFO score > 0.8) were kept. Ancestral outliers (*N* = 71, as defined by being >5 standard deviations from European samples in principal components 1 and 2), were not excluded. The LASER suit was used to infer ancestry (Taliun et al., 2017).

### Measures

#### Questionnaire-based measures for sleep, well-being, and subjective cognitive functioning

The sleep questions assessed total sleep duration, from which *long* and *short SD* were calculated, difficulties initiating sleep (*DIS*), early morning awakenings (*EMAs*), fatigue (*FAT*), and poor sleep quality (*poor SQ*) (for questions and response categories, see Table 1) (Cederlöf et al., 2022). The questions were based on Finnish general population studies (Aromaa & Koskinen, 2004; Heistaro, 2008; Partinen & Gislason, 1995), and for subjective well-being, the measures included *subjective health* (Heistaro, 2008), health-related quality of life (assessed with EQ-5D-3L, hereafter referred to as *EQ5D*) (Devlin & Krabbe, 2013; Saarni et al., 2006), *psychological distress* (assessed with the Mental Health Inventory–5 [*MHI-5*], according to RAND scoring instructions) (Aalto, Aro, Aro, Mähönen, & Aro, 1995; Berwick et al., 1991). Questions of *subjective cognitive functioning* included questions on concentration, learning, and memory (Heistaro, 2008; Koponen, Borodulin, Lundqvist, Sääksjärvi, & Koskinen, 2018). Poor subjective cognitive functioning was defined as giving the response poor or very poor to any of the three questions, a dummy variable previously used in Finnish population-based studies (Koponen et al., 2018).

**Table 1.** Questions used in the study

Questions	Answers	Categorized as
*Total sleep duration, TSD*: How long do you usually sleep at night? How long do you usually sleep during the daytime?	The patient wrote the number of hours and minutes for both questions freely. The night and day sleep were summed into TSD and compared with the Health 2000 study question ‘How many hours do you sleep per day?’	Responses that had missing or unreasonable answers to the sleep duration question (TSD < 2 hours or ≥ 18 hours) were excluded. Long SD was defined as sleeping 10 hours or more and short SD as sleeping 6 hours or less.
*Difficulty initiating sleep, DIS*: Do you have difficulties falling asleep without sleep medication?	Not at all, sometimes, often, and nearly always.	DIS was defined as ‘often’ and ‘nearly always’.
*Early morning awakenings, EMAs*: Do you wake up during sleep at night or very early in the morning?	Not at all, sometimes, often, and nearly always.	EMAs were defined as ‘often’ and ‘nearly always’.
*Fatigue, FAT*: Do you consider yourself to be fatigued more often than other people of the same age during the daytime?	Yes, nearly always; yes, often (at least weekly); no; I cannot say.	FAT was defined as ‘Yes, nearly always’ and ‘yes, often’.
*Sleep quality, SQ*: How well have you slept during the last month?	Well, rather well, neither well nor poorly, rather poorly, poorly.	Poor SQ was defined as having slept ‘rather poorly’ or ‘poorly’.
*Subjective health*: Assess your own current state of health by entering the number that best corresponds to your current state of health.	Numeric scale from 0 to 10.	—
*Health-related quality of life, EQ5D* Please tick the one box that best describes your health today.	3-level answer categories for mobility, self-care, usual activities, pain/discomfort, and anxiety/depression	Converted to a linear 0–1 variable based on country-specific conversion factors
*Mental Health Inventory–5, MHI–5* Please tick the one box that best describes your health today.	5 questions on mental health and psychological distress	The scores from the five questions were converted, added together, and used as a linear variable.
*Subjective cognitive functioning* *Focus:* How well are you usually able to focus? *Memory:* How do you rate how your memory functions? *Learning:* How do you rate your ability to learn new things?	Very well, well, satisfactorily, poorly, and very poorly	Poor subjective focus, memory, or learning was defined as ‘poorly’ or ‘very poorly’. A combined dummy variable named ‘poor subjective cognition’ was created which was defined as having answered ‘poorly’ or ‘very poorly in any of the three questions.

#### Assessment of objective cognitive functioning

Two tests were used: the *Reaction Time (RTI)* and the *Paired Associative Learning (PAL)* task, both from the Cambridge Neuropsychological Test Automated Battery (CANTAB). Five-choice serial reaction time task (5-CRTT) measured RTI to an unpredictable stimulus and hence general alertness and processing speed, while PAL measured visual and episodic learning and memory (CANTAB).

Our outcome for the RTI test was the median five-choice reaction time (RTIFMDRT), that is the median latency of response. The variable was calculated for correct responses where the stimulus could appear in any of the five locations. In the PAL test, our outcome was the Total Errors Adjusted (PALTEA), which reflects how quickly the participant learns when the participant has multiple attempts at each problem.

#### Interview

Marital status (marriage, common-law marriage, registered partnership), living status (unsupported versus supported housing), *participation in the workforce* (full- or part-time work, or student status), and education (register data obtained when interview data was unavailable) were asked in the interview.

*Clozapine use* was applied as an indicator of disease severity, as it is typically used in treatment-resistant psychotic disorders (Elkis & Buckley, 2016). Information on medications was retrieved from a question in the interview: ‘What medications do you use regularly?’ The names of the medicines were checked on the label or the prescription form unless the interviewee remembered them.

### FinnGen cohort

The FinnGen cohort data was used for analyzing the distribution of PGSs across the diagnostic groups and the general population. FinnGen is a public-private partnership, which combines genome information and digital healthcare data (Kurki et al., 2023). In this study, we used FinnGen Release 8, which comprises 356,077 Finnish individuals. We categorized the sample, using the same ICD-codes as in SUPER, into people with (1) schizophrenia (*N* = 6,280), (2) schizoaffective, bipolar disorder, or psychotic depression (*N* = 8,177), and (3) no psychiatric disorder (*N* = 251,638). Exactly 4,708 patients with schizophrenia and 3,511 patients with affective psychotic disorders were also included in the SUPER study. PGSs in FinnGen were calculated with the PRS-CS method (Ge et al., 2019). A total of 3,511 patients were found both in the FinnGen and the SUPER sample, but only 3,078 patients in SUPER (before applying inclusion criteria) had a diagnosis of schizoaffective disorder, bipolar disorder, or psychotic depression. This is likely due to diagnoses used in drug purchases and reimbursements being available in FinnGen, while only Finnish Care Register for Health Care (HILMO) diagnoses are available in the SUPER study. HILMO recognizes mostly only diagnoses made in the public health care system of Finland. An additional difference between the samples is that SUPER recruited patients with at least one psychotic episode in their medical history, and this does not apply to patients with bipolar disorder in the FinnGen study. Hence, this group from the FinnGen sample is referred to as ‘affective psychotic and bipolar disorders’.

### Statistical analysis

For analyzing associations between PGSs and outcomes, we conducted regression analyses in SPSS version 29.0, with age, gender, diagnostic group (schizophrenia and affective psychotic disorders), the first four principal components to control for population stratification, and one PGS value as independent variables. Logistic regression was conducted for the dichotomized dependent variables of sleep traits, subjective cognitive functioning, clozapine use, and work status, and linear regression was performed for MHI-5, EQ5D, RT, PAL, and days in involuntary hospitalization. We used a Bonferroni–Holm correction (0.05/14 [number of outcomes] = 0.0036 as the lowest significant threshold) to correct for multiple analyses (Aickin & Gensler, 1996). In supplemental analyses, we conducted the same analyses separately in the two diagnostic groups. Nagelkerke’s *R*^2^ was used to assess the proportion of variance explained by PGS_SD_ and PGS_INS_ (Nagelkerke, 1991).

To analyze the distribution of the PGSs in the FinnGen cohort, we conducted binomial regression analyses in R version 4.1.3. The first four principal components and gender were included as covariates. The PGS was the independent variable, while the diagnosis was the dependent variable. A Bonferroni–Holm correction (0.05/6 [amount of PGSs] = 0.0083) was used to correct for multiple analyses. The regression analyses were conducted separately for the two diagnostic groups, and people with no psychiatric disorders were the reference group. We replicated the full FinnGen sample analyses, with the two subsamples of (1) patients who were a part of both FinnGen and the SUPER study, and (2) the patients in FinnGen who were not in the SUPER study.

## Results

### Demographics

The largest diagnostic group was patients with schizophrenia (demographics in Table 2). Patients with schizophrenia had on average a longer time since the first diagnosis of psychosis (22.0 vs. 12.9 years), lower level of education, and lower participation in the workforce than patients with affective psychotic disorders.

**Table 2.** Demographics of the SUPER study

	SZ	Affective psychotic disorders (SZ-A, BPD, and Ps-DEP)
Proportion of sample, *n* (%)	5353 (65.0)	2879 (35.0)
Gender (Men), *n* (%)	3069 (57.3)	1079 (37.5)
18–40 years, *n* (%)	1674 (31.3)	1185 (41.2)
41–60 years, *n* (%)	2460 (46.0)	1210 (42.0)
61–80 years, *n* (%)	1219 (22.8)	484 (16.8)
Age, *M* (SD)	48.3 (13.9)	45.1 (14.6)
Years since onset, *M* (SD)	22.0 (12.8)	12.9 (8.9)
Low level of education, *n* (%)	2166 (40.5)	656 (22.8)
Intermediate level of education, *n* (%)	2345 (43.8)	1381 (48.0)
High level of education, *n* (%)	820 (15.3)	839 (29.1)
Married/cohabiting, *n* (%)	695 (13.0)	952 (33.5)
Participation in workforce	395 (7.7)	507 (18.4)
Living independently	3151 (59.5)	2477 (87.1)
Clozapine medication	2014 (38.3)	233 (8.4)

*Note:* SZ, schizophrenia; SZ-A, schizoaffective disorder; BD, bipolar disorder; Ps-DEP, psychotic depression.

### Sleep-related measures

**Figure 1.**
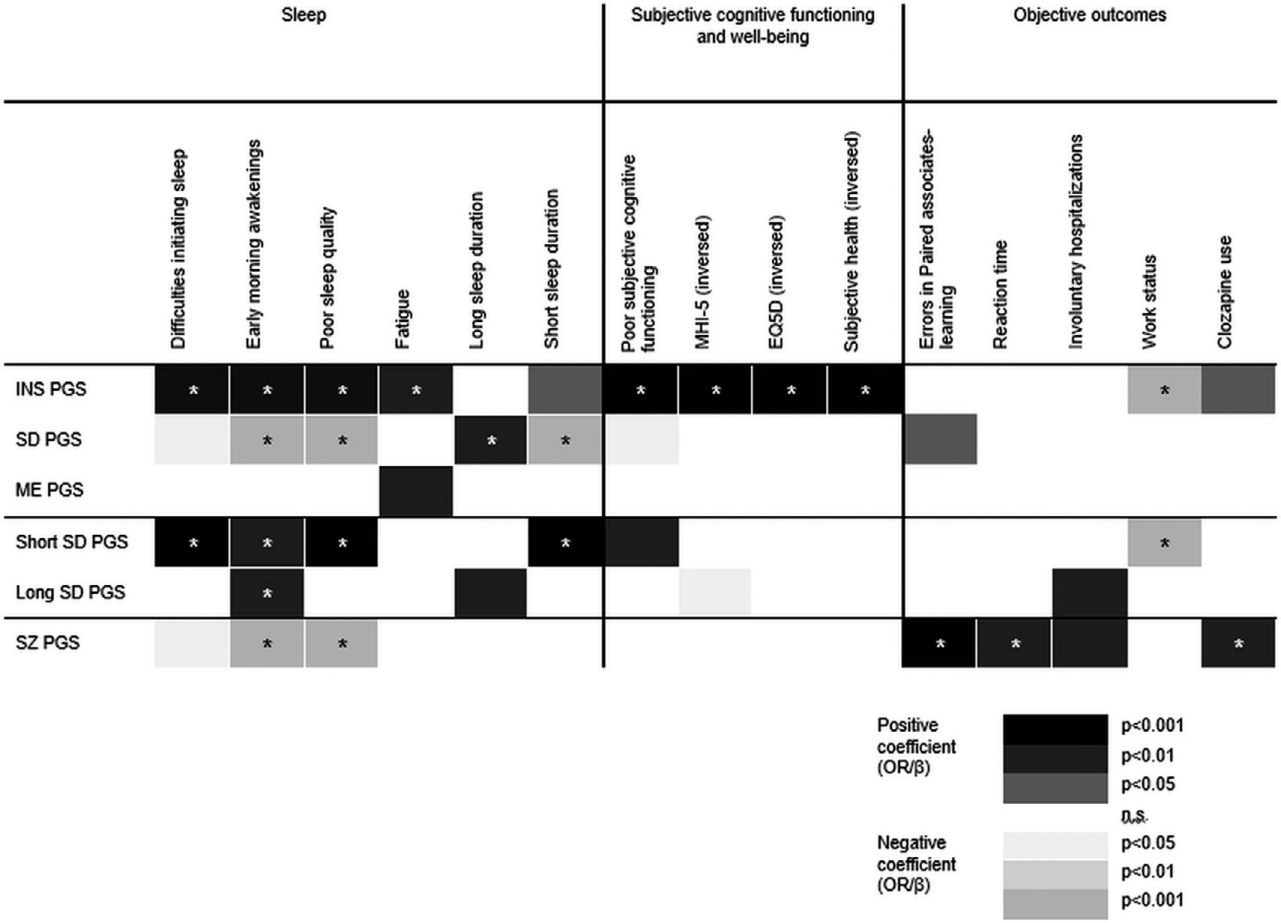
Results from logistic and linear regression analyses for the polygenic scores. For congruency, the outcomes of subjective health, MHI-5, and EQ5D were inversed for this figure. Darker gray indicates positive coefficients (representing a worse outcome), while lighter gray indicates negative coefficients. * = significant after Bonferroni–Holm correction. All results significant before correction (*p* < 0.05) are colored. *Note*: PGSs in rows: INS, ‘insomnia’; SD, ‘sleep duration’; ME, ‘morning-eveningness (chronotype)’; SZ, ‘schizophrenia’; MHI-5, ‘Mental Health Inventory -5’; EQ5D, ‘Health-related quality of life’.

Using polygenic scores for sleep traits, diurnal preference (eveningness), and schizophrenia, we investigated how the polygenic burden was associated with sleep problems within the psychotic disorder spectrum (Figure 1 and Supplementary Table 1). PGS_INS_, PGS_SD_, and PGS_SS_ showed the strongest impact on sleep. After Bonferroni–Holm correction, high PGS_INS_ was significantly associated with DIS (OR = 1.18, 95 % CI = 1.12–1.24, *p* = 7.12 × 10^−11^), EMAs (OR = 1.11, CI = 1.06–1.17, *p* = 6.60 × 10^−6^), poor SQ (OR = 1.22, CI = 1.15–1.30, *p* = 8.68 × 10^−10^), and FAT (OR = 1.08, CI = 1.03–1.13, *p* = .002), and similar findings were observed with PGS_SS_ (DIS, OR = 1.18, CI = 1.05–1.17, *p* = 7.20 × 10^−5^; EMAs, OR = 1.08, CI = 1.03–1.13, *p* = .001; short SD, OR = 1.18, CI = 1.09–1.27, *p* = 6.15 × 10^−5^; poor SQ, OR = 1.18, CI = 1.11–1.26, *p* = 4.53 × 10^−4^). High PGS_SD_ was associated with less EMAs (OR = 0.92, CI = 0.87–0.96, *p* = 4.41 × 10^−4^), better SQ (OR = 0.87, CI = 0.82–0.93, *p* = 3.87 × 10^−5^), and with longer SD (short SD, OR = 0.86, CI = 0.79–0.93, *p* = 1.83 × 10^−4^; long SD, OR = 1.09, CI = 1.03–1.14, *p* = .001). PGS_LS_ was only associated with more EMAs (OR = 1.07, CI = 1.02–1.12, *p* = .003) and PGS_ME_ was not significantly associated with any of the sleep outcomes. High PGS_SZ_ was associated with less EMAs (OR = 0.90, CI = 0.86–0.95, *p* = 2.34 × 10^−4^) and better sleep quality (poor SQ, OR = 0.87, CI = 0.81–0.94, *p* = 2.88 × 10^−4^). In a linear regression analysis of sleep duration, PGS_SD_ explained 0.3% of the variation in phenotypic sleep duration, while 0.9% of poor SQ variance, as measured by Nagelkerke *R*^2^, was explained by PGS_INS_.

### Well-being and subjective cognitive functioning

PGS_INS_ was the only PGS with significant associations (Figure 1 and Supplementary Tables 2 and 3), and the score was associated with all non-sleep subjective measures, including poorer subjective cognitive functioning (OR = 1.14, CI = 1.09–1.20, *p* = 1.86 × 10^–7^), poorer subjective health, β = −0.06, CI = −0.08, −0.03, *p* = 5.23 × 10^−7^), worse EQ5D (β = −0.07, CI = −0.09, -0.05, *p* = 1.89 × 10^−9^) and MHI-5 (β = −0.06, CI = −0.08, −0.02, *p* = 4.05 × 10^−8^).

### Cognitive tests and measures for disease severity

Mainly PGS_SZ_ had significant associations with objective outcomes (Figure 1 and Supplementary Table 4), including worse results in PAL (β = 0.07, CI = 0.04, 0.09, *p* = 1.60 × 10^–6^) and RTI (β = 0.05, CI = 0.02, 0.07, *p* = .001), and more frequent clozapine use (OR = 1.15, CI = 1.08–1.22, *p* = 6.13 × 10^−7^). PGS_INS_, and PGS_SS_ were associated with a lower risk of unemployment (OR = 0.89, CI = 0.83–0.96, *p* = .003, and OR = 0.88, CI = 0.82–0.95, *p* = .002, respectively).

In analyses with diagnostic splits (Supplementary Table 7), the association between high PGS_SZ_ and worse performance in PAL and RTI remained significant only in patients with affective psychotic disorders.

### Distribution of PGSs across the diagnostic spectrum and the general population

**Figure 2.**
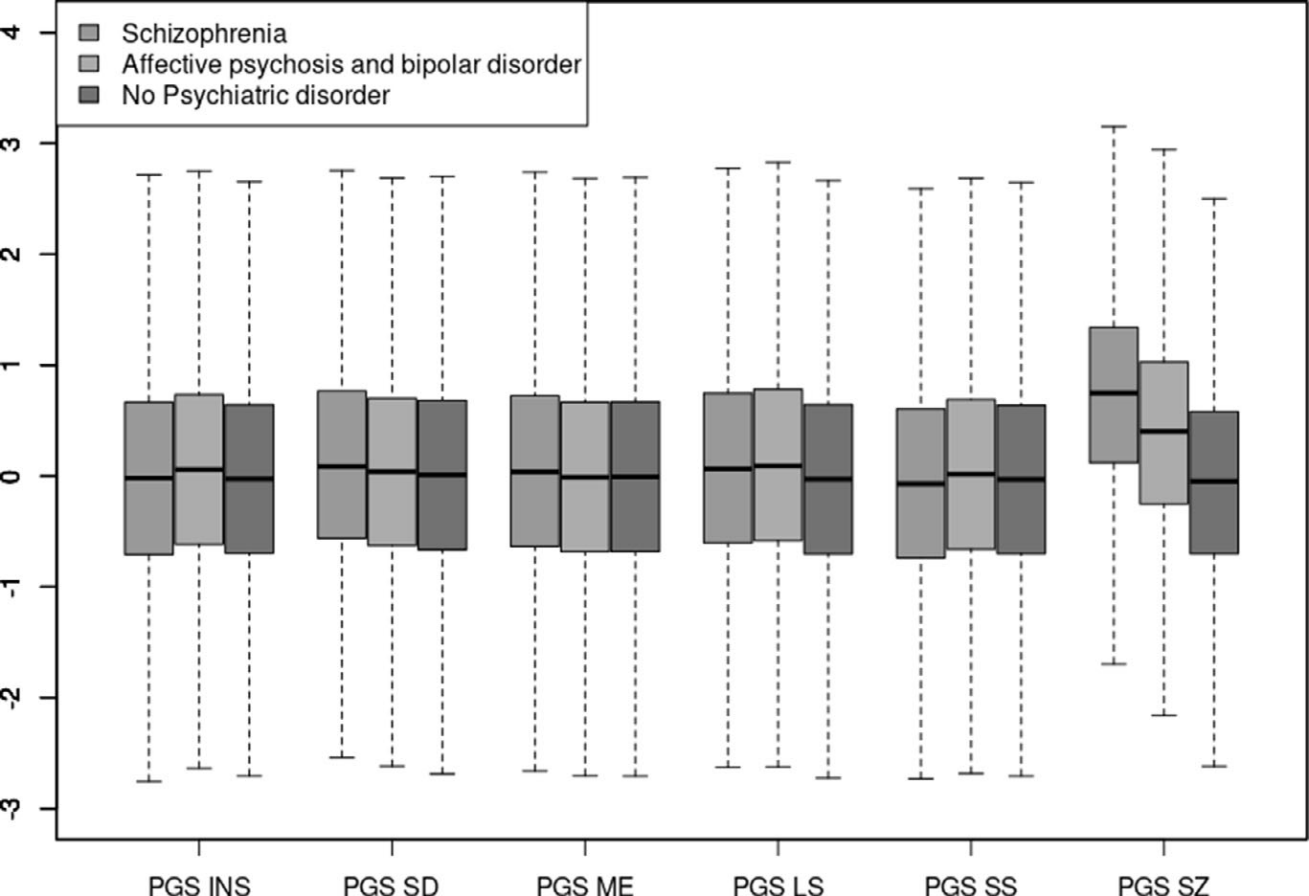
Boxplot showing the distribution of polygenic scores across different diagnostic groups.

We investigated the difference in the distribution of PGSs in diagnostic groups (schizophrenia and affective psychotic and bipolar disorders) compared with people with no psychiatric disorders (Figure 2 for boxplot, Table 3). When analyzing the full FinnGen sample, patients with schizophrenia had a very strong association with PGS_SZ_, and associations with PGS_SD_ (OR = 1.09, CI = 1.06–1.11, *p* = 1 × 10^−10^) and PGS_LS_ (OR = 1.09, CI = 1.06–1.12, *p* = 2 × 10^−11^), and associations with PGS_ME_ (OR = 1.06, CI = 1.04–1.09, *p* = 2 × 10^−6^), and PGS_SS_ (OR = 0.96, CI = 0.92–0.98, *p* = .002). For patients with affective bipolar and psychotic disorders, there were associations with PGS_SZ_, PGS_INS_ (OR = 1.08, CI = 1.05–1.10, *p* = 3 × 10^−12^), PGS_LS_ (OR = 1.12, CI = 1.09–1.14, *p* < 2 × 10^−16^), and an association with PGS_SS_ (OR = 1.05, CI = 1.03–1.07, 1 × 10^−5^).

**Table 3.** Results from linear regression analyses of PGSs in the diagnostic groups, compared with people with no psychiatric disorder

Outcome diagnosis	Schizophrenia PGS			Sleep duration PGS			Insomnia PGS			Eveningness PGS			Long sleep duration PGS			Short sleep duration PGS		
Full Finngen sample	OR	CI	*p*	OR	CI	*p*	OR	CI	*p*	OR	CI	*p*	OR	CI	*p*	OR	CI	*p*
Schizophrenia (*N* = 6,280)	2.39	2.32–2.46	< 2 × 10^−16^	1.09	1.06–1.11	1 × 10^−10^	1.00	0.97–1.02	.698	1.06	1.04–1.09	2 × 10^−6^	1.09	1.06–1.12	2 × 10^−11^	0.96	0.94–0.98	.002
Affective psychotic and bipolar disorders (*N* = 9,563)	1.64	1.60–1.67	< 2 × 10^−16^	1.02	1.00–1.05	.026	1.08	1.05–1.10	3 × 10^−12^	1.01	0.99–1.03	.319	1.12	1.09–1.14	< 2 × 10^−16^	1.05	1.03–1.07	1 × 10^−5^

A separate analysis of (1) SUPER patients and (2) patients only in FinnGen (Supplementary Table 8), found similar patterns of associations in both data sets for PGS_SZ_. In SUPER, PGS_SD_ was associated with affective psychotic disorders, as did PGS_ME_ with marginal statistical evidence. These associations were not found in Finngen, or in the complete data set. PGS_SZ_ was higher in the SUPER sample than in the FinnGen sample.

## Discussion

To the best of our knowledge, this is the first systematic study on polygenic influences for sleep traits in psychotic disorders. We found that sleep PGSs, derived from the general population, are significantly associated with sleep traits also in this population with psychiatric comorbidity and high rates of sleep-impacting medications (Cederlöf et al., 2024). Additionally, we identified varied distributions of sleep PGSs across non-affective and affective psychotic disorders and individuals with no psychiatric disorder. A high genetic risk for insomnia was consistently associated with worse well-being, while that for schizophrenia had an impact on objective measures, including poorer performance in cognitive tests and more clozapine use.

Different types of sleep problems have been previously linked to psychotic disorders in clinical studies, where they are often considered secondary to the disorder or medication (Krystal, 2012), albeit our findings in non-medicated patients show that they also present more frequently with both insomnia and hypersomnia symptoms than the general population (Cederlöf et al., 2022, 2024). Our present finding on the association of sleep PGSs to sleep traits in psychotic disorders shows how intrinsic genetic vulnerability for insomnia independently links to sleep problems. On the other hand, the lack of association of the sleep PGSs to any of the objective measures was striking. For example, while PGS_INS_ was associated significantly with subjective experience of impaired cognitive functioning, it was not correlated with the objective measures of cognition (vigilance or verbal learning). Contrary to this, PGS_SZ_ was not linked to subjective but only to worse objective measures on cognitive functions, potentially linked to sleep spindle and slow-wave abnormalities observed in earlier studies of patients with schizophrenia (Ferrarelli, 2021). Furthermore, PGS_SZ_ correlated negatively with subjective experiences of sleep problems. Thus, according to these findings, sleep symptoms seem to form a separate domain of symptoms with distinctive etiological influences among patients with psychotic disorders.

In our attempt to find differences in genetic influences on non-affective and affective psychosis, we found a consistent pattern of deviating risks in both datasets, the SUPER sample and the FinnGen data. As expected, PGS_SZ_ is associated robustly with schizophrenia, showing a > 2-fold risk compared with the general population (Allardyce et al., 2018; Sullivan et al., 2018). It was also elevated in affective psychotic and bipolar disorders but with a lower OR. PGS_INS_ was significantly higher among patients with affective psychotic disorders, but not among those with schizophrenia, as compared with the general population. This finding supports the previously well-known role of insomnia as a risk factor for affective disorders (Hertenstein, Benz, Schneider, & Baglioni, 2023).

Regarding the three PGSs related to sleep duration, we observed a pattern in which patients with schizophrenia had higher levels of the PGS_SD_ and PGS_LS_ scores (reflecting genetic propensities for long sleep duration), while patients with affective psychotic and bipolar disorders had elevated levels of both PGS_LS_ and PGS_SS_ (reflecting genetic propensities for both short and long sleep duration). The finding on schizophrenia supports the role of genetic influences in our earlier observation of longer sleep duration in psychotic disorders compared with the general population (Cederlöf et al., 2022). Comprehensively, our findings provide evidence of distinct patterns of genetic sleep-related risks in non-affective and affective psychosis.

Previous studies have proposed diurnal preference for eveningness as a risk for compromised mental health, in particular depression (Norbury, 2021), and according to some studies, schizophrenia and bipolar disorder as well (Linke & Jankowski, 2021). In this study, PGS_ME_ was not associated significantly with any subjective or objective measures among the SUPER patients. In the full FinnGen data, there was, however, a significant and relatively robust correlation to schizophrenia but not to affective psychotic and bipolar disorders. One potential mechanism underlying eveningness-type of diurnal preference is a slower dynamic of sleep pressure build-up and dissipation(Mongrain & Dumont, 2007; Taillard, Philip, Coste, Sagaspe, & Bioulac, 2003). Our findings for PGS_SD_ and PGSME suggest the presence of deviating processes in homeostatic sleep regulation in schizophrenia – a conclusion that would be in line with the previous studies indicating slow-wave sleep deficits in schizophrenia (Ferrarelli, 2021).

*Strengths and limitations.* Our study comprised a large nationwide sample of patients with psychotic disorders, combining genetic information with rich phenotype data including extensive subjective metrics and cognitive tests. The use of the FinnGen database enabled novel large-sample analyses on sleep PGSs in the different diagnostic groups. There are, however, some limitations that should be considered when interpreting the findings. It is important to note that these findings were derived from a relatively homogenous population, which can be advantageous for some of the exploratory investigations presented in this article; however, this homogeneity may also limit the generalizability of the results (Woodward, Urbanowicz, Naj, & Moore, 2022). The impact of sleep PGSs was modest on sleep phenotypes, in line with previous studies in the general population and patients with major depressive disorder (Dashti, Jones, et al., 2019; Melhuish Melhuish Beaupre et al., 2021). Second, self-assessment of cognition and sleep is known to be deficient in patients with psychotic disorders, as factors such as depressed mood can exaggerate symptoms (Demant, Vinberg, Kessing, & Miskowiak, 2015; Gonzalez, Tamminga, Tohen, & Suppes, 2013; Raffard, Lebrun, Bayard, Macgregor, & Capdevielle, 2020), while simultaneously some patients may have limited awareness of their cognitive deficits (Homayoun, Nadeau-Marcotte, Luck, & Stip, 2011; Prouteau, Roux, Destaillats, & Bergua, 2017). Moreover, the cognitive measures could also be more extensive and include domains such as executive functions or social cognition (CANTAB). Finally, we did not have objective sleep data in the present study. Such data would be valuable not only in considering the modest effect of sleep PGSs on objective sleep in the general population (Dashti, Jones, et al., 2019; Foldager et al., 2022), but also in further elucidating potential pathogenic sleep-related processes in psychotic disorders.

## Conclusions

Sleep PGSs derived from the general population influence self-reported sleep also in persons with psychotic disorders. PGS_INS_ is associated with worse subjective well-being, but not with cognitive deficits or other objective measures of disease severity, while PGS_SZ_ is related to worse objective measures, but not to worse subjective measures. Our findings underscore the importance of sleep for the quality of life of patients, and the etiologic heterogeneity between core objective elements of psychotic disorders and the subjective measures related to sleep and well-being.

## Supporting information

Cederlöf et al. supplementary material Cederlöf et al. supplementary material

## Supplementary material

The supplementary material for this article can be found at http://doi.org/10.1017/S0033291725000844.
