# Supplementary material for: Sleep and schizophrenia polygenic scores in non-affective and affective psychotic disorders
Source: Psychol Med. 2025 Apr 15;55:e117. doi: 10.1017/S0033291725000844 (PMC12094621; doi:10.1017/S0033291725000844)
Supplement: Cederlöf et al. supplementary material [file S0033291725000844sup001.docx]

**Supplementary Table 1-4.** Results from logistic and linear regression analyses, that are included in Figure 1 (heatmap) in the article.

**Supplementary Table 5-7.** Results from logistic and linear regression subanalyses, with diagnostic or age group split.

**Supplementary Table 8.** Results from linear regression analyses of PGSs in the diagnostic groups, compared to people with no psychiatric disorder, in the two subgroups of SUPER patients in Finngen sample, and Finngen sample without SUPER patients.

**Supplementary Figure 1.** STROBE Flowchart of SUPER study sample used in this study.

**Supplementary table 1.** PGSs association to sleep outcomes in sample.

|  | **OR** | **CI** | **p** |
| --- | --- | --- | --- |
| **Difficulties initiating sleep** | | | |
| Insomnia PGS | **1.18** | **1.12-1.24** | **7.12×10^-11^** |
| Sleep duration PGS | 0.94 | 0.88-0.99 | .022 |
| Eveningness PGS | 1.04 | 0.99-1.10 | .088 |
| Schizophrenia PGS | 0.94 | 0.89-1.00 | .037 |
| Short SD PGS | **1.11** | **1.05-1.17** | **7.20×10^-5^** |
| Long SD PGS | 1.03 | 0.98-1.08 | .225 |
| **Early morning awakenings** | | | |
| Insomnia PGS | **1.11** | **1.06-1.17** | **6.60×10^-6^** |
| Sleep duration PGS | **0.92** | **0.87-0.96** | **4.41×10^-4^** |
| Eveningness PGS | 0.98 | 0.94-1.03 | .412 |
| Schizophrenia PGS | **0.90** | **0.86-0.95** | **2.34×10^-4^** |
| Short SD PGS | **1.08** | **1.03-1.13** | **.001** |
| Long SD PGS | **1.07** | **1.02-1.12** | **.003** |
| **Fatigue** |  |  |  |
| Insomnia PGS | **1.08** | **1.03-1.13** | **.002** |
| Sleep duration PGS | 0.97 | 0.92-1.01 | .167 |
| Eveningness PGS | 1.07 | 1.02-1.12 | .007 |
| Schizophrenia PGS | 0.96 | 0.91-1.01 | .116 |
| Short SD PGS | 1.01 | 0.97-1.06 | .575 |
| Long SD PGS | 1.00 | 0.96-1.05 | .997 |
| **Short sleep duration** | | | |
| Insomnia PGS | 1.11 | 1.03-1.20 | .009 |
| Sleep duration PGS | **0.86** | **0.79-0.93** | **1.83×10^-4^** |
| Eveningness PGS | 1.00 | 0.92-1.08 | .935 |
| Schizophrenia PGS | 0.94 | 0.86-1.02 | .151 |
| Short SD PGS | **1.18** | **1.09-1.27** | **6.15×10^-5^** |
| Long SD PGS | 0.99 | 0.91-1.07 | .745 |
| **Long sleep duration** | | | |
| Insomnia PGS | 0.99 | 0.95-1.04 | .772 |
| Sleep duration PGS | **1.09** | **1.03-1.14** | **.001** |
| Eveningness PGS | 1.03 | 0.98-1.08 | .232 |
| Schizophrenia PGS | 1.05 | 0.99-1.11 | .119 |
| Short SD PGS | 1.00 | 0.95-1.05 | .855 |
| Long SD PGS | 1.07 | 1.02-1.12 | .009 |
| **Poor sleep quality** | | | |
| Insomnia PGS | **1.22** | **1.15-1.30** | **8.68×10^-10^** |
| Sleep duration PGS | **0.87** | **0.82-0.93** | **3.87×10^-5^** |
| Eveningness PGS | 1.01 | 0.95-1.08 | .780 |
| Schizophrenia PGS | **0.87** | **0.81-0.94** | **2.88×10^-4^** |
| Short SD PGS | **1.18** | **1.11-1.26** | **4.53×10^-4^** |
| Long SD PGS | 1.04 | 0.97-1.10 | .288 |

|  | **β** | **CI** | **p** |
| --- | --- | --- | --- |
| **Subjective health** | | | |
| Insomnia PGS | **-0.06** | **-0.08, -0.03** | **5.23×10^-7^** |
| Sleep duration PGS | 0.02 | -0.01, 0.04 | .157 |
| Eveningness PGS | 0.01 | -0.01, 0.03 | .397 |
| Schizophrenia PGS | 0.02 | -0.01, 0.04 | .189 |
| Short SD PGS | -0.02 | -0.04, 0.00 | .102 |
| Long SD PGS | -0.01 | -0.03, 0.02 | .537 |
| **MHI-5** | | | |
| Insomnia PGS | **-0.06** | **-0.08, -0.04** | **4.05×10^-8^** |
| Sleep duration PGS | 0.02 | -0.004, 0.04 | .106 |
| Eveningness PGS | -0.004 | -0.03, 0.02 | .736 |
| Schizophrenia PGS | 0.02 | -0.01, 0.04 | .224 |
| Short SD PGS | -0.02 | -0.04, 0.01 | .189 |
| Long SD PGS | -0.02 | -0.05, -0.001 | .037 |
| **EQ5D** |  |  |  |
| Insomnia PGS | **-0.07** | **-0.09, -0.05** | **1.89×10^-9^** |
| Sleep duration PGS | 0.01 | -0.01, 0.04 | .315 |
| Eveningness PGS | -0.003 | -0.03, 0.02 | .766 |
| Schizophrenia PGS | 0.002 | -0.02, 0.03 | .892 |
| Short SD PGS | -0.02 | -0.04, 0.01 | .201 |
| Long SD PGS | -0.02 | -0.04, 0.002 | .073 |

**Supplementary table 2.** PGSs association to subjective health, MHI-5, EQ5D

**Supplementary table 3.** PGSs association to three domains (attention, learning, memory) of poor subjective cognitive functioning and to the dummy variable of those three combined.

|  | **OR** | **CI** | **p** |
| --- | --- | --- | --- |
| **Memory** | | | |
| Insomnia PGS | **1.15** | **1.09–1.23** | **3.88×10^–6^** |
| Sleep duration PGS | 0.96 | 0.90–1.02 | .191 |
| Eveningness PGS | 0.98 | 0.92–1.04 | .535 |
| Schizophrenia PGS | 0.94 | 0.88–1.01 | .100 |
| Short SD PGS | 1.07 | 1.01–1.14 | .025 |
| Long SD PGS | 1.07 | 1.01–1.14 | .030 |
| **Learning** | | | |
| Insomnia PGS | **1.11** | **1.04–1.18** | **7.98×10^–4^** |
| Sleep duration PGS | 0.96 | 0.90–1.02 | .175 |
| Eveningness PGS | 1.01 | 0.95–1.07 | .815 |
| Schizophrenia PGS | 1.01 | 0.94–1.08 | .782 |
| Short SD PGS | 1.04 | 0.98–1.10 | .222 |
| Long SD PGS | 1.05 | 0.98–1.11 | .149 |
| **Focus** |  |  |  |
| Insomnia PGS | **1.13** | **1.06–1.20** | **1.13×10^–4^** |
| Sleep duration PGS | 0.93 | 0.87–0.99 | .019 |
| Eveningness PGS | 1.03 | 0.97–1.09 | .393 |
| Schizophrenia PGS | 1.01 | 0.94–1.08 | .877 |
| Short SD PGS | 1.07 | 1.00–1.14 | .043 |
| Long SD PGS | 0.98 | 0.92–1.04 | .505 |
| **Any** | | | |
| Insomnia PGS | **1.14** | **1.09–1.20** | **1.86×10^–7^** |
| Sleep duration PGS | 0.94 | 0.90–0.99 | .026 |
| Eveningness PGS | 0.99 | 0.94–1.04 | .688 |
| Schizophrenia PGS | 0.98 | 0.92–1.03 | .394 |
| Short SD PGS | 1.08 | 1.02–1.13 | .004 |
| Long SD PGS | 1.022 | 0.97–1.07 | .388 |

**Supplementary table 4.** PGSs association to objective outcomes, meaning, paired-associated learning test (PAL), median reaction time (RT), days in involuntary hospitalization and work status. PAL and work status were analyzed in logistic regression models, reaction time and involuntary hospitalizations in linear regression models.

|  | **β/OR** | **CI** | **p** |
| --- | --- | --- | --- |
| **PAL** | | | |
| Insomnia PGS | 0.01 | -0.01, 0.04 | .320 |
| Sleep duration PGS | 0.03 | 0.003, 0.05 | .025 |
| Eveningness PGS | -0.01 | -0.03, 0.01 | .422 |
| Schizophrenia PGS | **0.07** | **0.04, 0.09** | **1.60×10^–6^** |
| Short SD PGS | 0.03 | 0.002, 0.05 | .037 |
| Long SD PGS | 0.02 | -0.01, 0.04 | .152 |
| **RT** | | | |
| Insomnia PGS | -0.02 | -0.04, 0.003 | .085 |
| Sleep duration PGS | 0.01 | -0.02, 0.03 | .655 |
| Eveningness PGS | -0.01 | -0.03, 0.02 | .676 |
| Schizophrenia PGS | 0.05 | 0.02, 0.07 | .001 |
| Short SD PGS | -0.01 | -0.04, 0.01 | .382 |
| Long SD PGS | 0.002 | -0.021, 0.026 | .854 |
| **Involuntary hospitalizations** |  |  |  |
| Insomnia PGS | 0.01 | -0.01, 0.04 | .239 |
| Sleep duration PGS | 0.002 | -0.02, 0.02 | .876 |
| Eveningness PGS | -0.004 | -0.03, 0.02 | .752 |
| Schizophrenia PGS | 0.03 | 0.01, 0.06 | .009 |
| Short SD PGS | 0.02 | -0.01, 0.04 | .139 |
| Long SD PGS | 0.03 | 0.01, 0.05 | .005 |
| **Work status** | | | |
| Insomnia PGS | **0.89** | **0.83-0.96** | **.003** |
| Sleep duration PGS | 1.06 | 0.98-1.15 | .122 |
| Eveningness PGS | 0.94 | 0.87-1.01 | .091 |
| Schizophrenia PGS | 0.93 | 0.85-1.01 | .101 |
| Short SD PGS | **0.88** | **0.82-0.95** | **.002** |
| Long SD PGS | 0.96 | 0.89-1.04 | .307 |
| **Clozapine use** |  |  |  |
| Insomnia PGS | 1.06 | 1.01-1.12 | .023 |
| Sleep duration PGS | 1.01 | 0.95-1.06 | .837 |
| Eveningness PGS | 1.03 | 0.98-1.09 | .219 |
| Schizophrenia PGS | **1.15** | **1.08-1.22** | **6.13×10^-7^** |
| Short SD PGS | 1.04 | 0.99-1.10 | .152 |
| Long SD PGS | 1.04 | 0.99-1.10 | .135 |

**Supplementary Table 5.** Association between polygenic risk scores and sleep outcomes in different diagnostic groups. Gender and age were included as covariates.

|  |  | **Schizophrenia** | | | **Affective psychoses** | | |
| --- | --- | --- | --- | --- | --- | --- | --- |
|  |  | **OR** | **CI** | **p** | **OR** | **CI** | **p** |
| **Difficulties initiating sleep** | | | | | | | |
|  | Insomnia PGS | **1.19** | **1.11-1.27** | **<.001** | **1.17** | **1.09-1.27** | **<.001** |
|  | Sleep duration PGS | 0.91 | 0.85-0.97 | .005 | 0.98 | 0.91-1.06 | .668 |
|  | Eveningness PGS | 1.07 | 1.01-1.15 | .035 | 1.01 | 0.93-1.09 | .862 |
|  | Schizophrenia PGS | 0.97 | 0.90-1.04 | .389 | 0.90 | 0.82-0.98 | .018 |
|  | Short SD PGS | **1.11** | **1.04-1.19** | **.002** | 1.10 | 1.02-1.20 | .015 |
|  | Long SD PGS | 1.04 | 0.98-1.11 | .195 | 1.01 | 0.94-1.09 | .769 |
| **Early morning awakenings** | | | | | | | |
|  | Insomnia PGS | **1.15** | **1.08-1.22** | **<.001** | 1.06 | 0.98-1.15 | .123 |
|  | Sleep duration PGS | **0.91** | **0.86-0.97** | **.002** | 0.97 | 0.90-1.05 | .418 |
|  | Eveningness PGS | 0.96 | 0.91-1.02 | .189 | 1.01 | 0.94-1.10 | .728 |
|  | Schizophrenia PGS | 0.91 | 0.85-0.97 | .007 | 0.89 | 0.81-0.97 | .011 |
|  | Short SD PGS | 1.07 | 1.01-1.14 | .021 | 1.10 | 1.02-1.19 | .017 |
|  | Long SD PGS | 1.09 | 1.03-1.15 | .004 | 1.04 | 0.97-1.13 | .281 |
| **Fatigue** |  |  |  |  |  |  |  |
|  | Insomnia PGS | 1.07 | 1.01-1.13 | .023 | 1.10 | 1.01-1.18 | .020 |
|  | Sleep duration PGS | 0.96 | 0.90-1.01 | .128 | 0.98 | 0.91-1.06 | .641 |
|  | Eveningness PGS | 1.09 | 1.03-1.15 | .004 | 1.03 | 0.95-1.11 | .510 |
|  | Schizophrenia PGS | 0.97 | 0.90-1.03 | .311 | 0.94 | 0.86-1.03 | .166 |
|  | Short SD PGS | 1.01 | 0.95-1.07 | .843 | 1.03 | 0.95-1.12 | .465 |
|  | Long SD PGS | 0.98 | 0.93-1.04 | .561 | 1.03 | 0.95-1.12 | .443 |
| **Short sleep duration** | | | | | | | |
|  | Insomnia PGS | 1.11 | 1.00-1.24 | .045 | 1.11 | 0.99-1.25 | .069 |
|  | Sleep duration PGS | 0.86 | 0.77-0.96 | .005 | 0.86 | 0.76-0.97 | .014 |
|  | Eveningness PGS | 1.01 | 0.91-1.12 | .869 | 0.98 | 0.87-1.11 | .764 |
|  | Schizophrenia PGS | 0.92 | 0.81-1.03 | .159 | 0.96 | 0.83-1.09 | .510 |
|  | Short SD PGS | 1.09 | 0.98-1.21 | .125 | **1.31** | **1.16-1.48** | **<.001** |
|  | Long SD PGS | 1.01 | 0.91-1.12 | .829 | 0.96 | 0.85-1.08 | .470 |
| **Long sleep duration** | | | | | | | |
|  | Insomnia PGS | 0.97 | 0.91-1.03 | .694 | 1.04 | 0.95-1.13 | .400 |
|  | Sleep duration PGS | 1.09 | 1.03-1.16 | .004 | 1.05 | 0.97-1.15 | .232 |
|  | Eveningness PGS | 1.05 | 0.99-1.11 | .131 | 1.00 | 0.92-1.09 | .984 |
|  | Schizophrenia PGS | 1.05 | 0.98-1.13 | .144 | 1.04 | 0.94-1.14 | .494 |
|  | Short SD PGS | 0.99 | 0.94-1.06 | .832 | 1.00 | 0.91-1.09 | .927 |
|  | Long SD PGS | 1.07 | 1.01-1.13 | .026 | 1.06 | 0.98-1.16 | .169 |
| **Poor sleep quality** | | | | | | | |
|  | Insomnia PGS | **1.18** | **1.08-1.29** | **<.001** | **1.27** | **1.16-1.39** | **<.001** |
|  | Sleep duration PGS | **0.86** | **0.79-0.94** | **<.001** | 0.92 | 0.84-1.01 | .084 |
|  | Eveningness PGS | 1.03 | 0.94-1.12 | .817 | 0.99 | 0.90-1.08 | .817 |
|  | Schizophrenia PGS | 0.87 | 0.79-0.97 | .009 | 0.87 | 0.79-0.97 | .010 |
|  | Short SD PGS | 1.14 | 1.04-1.25 | .004 | **1.23** | **1.12-1.35** | **<.001** |
|  | Long SD PGS | 1.03 | 0.94-1.12 | .574 | 1.04 | 0.95-1.14 | .347 |

**Supplementary Table 6.** Association between polygenic risk scores and subjective and objective phenotypes in different diagnostic groups. Gender and age group were included as covariates.

|  |  | **Schizophrenia** | | | **Affective psychoses** | | |
| --- | --- | --- | --- | --- | --- | --- | --- |
|  |  | **β/OR** | **CI** | **p** | **β/OR** | **CI** | **p** |
| **Subjective health** | | | | | | | |
|  | Insomnia PGS | **-0.05** | **-0.08, -0.03** | **<.001** | **-0.07** | **-0.11, -0.03** | **<.001** |
|  | Sleep duration PGS | 0.02 | -0.01-0.05 | .115 | 0.02 | -0.02-0.06 | .402 |
|  | Eveningness PGS | 0.003 | -0.03, 0.03 | .830 | 0.02 | -0.02, 0.06 | .252 |
|  | Schizophrenia PGS | 0.001 | -0.03, 0.03 | .961 | 0.05 | 0.004, 0.09 | .033 |
|  | Short SD PGS |  |  |  |  |  |  |
|  | Long SD PGS | 0.01 | -0.02, 0.04 | .455 | -0.04 | -0.08, 0.00 | .049 |
| **EQ-5D** | | | | | | | |
|  | Insomnia PGS | **-0.05** | **-0.08, -0.02** | **<.001** | **-0.11** | **-0.15, -0.07** | **<.001** |
|  | Sleep duration PGS | 0.03 | 0.01-0.06 | .018 | 0.01 | -0.03-0.05 | .505 |
|  | Eveningness PGS | -0.02 | -0.04-0.01 | .282 | 0.02 | -0.02, 0.06 | .330 |
|  | Schizophrenia PGS | 0.01 | -0.03, 0.04 | .661 | 0.001 | -0.04, 0.05 | .948 |
|  | Short SD PGS | 0.01 | -0.02, 0.04 | .532 | -0.06 | -0.10, -0.02 | .003 |
|  | Long SD PGS | -0.01 | -0.04, 0.02 | .285 | -0.04 | -0.08, -0.01 | .022 |
| **MHI-5** | | | | | | | |
|  | Insomnia PGS | **-0.05** | **-0.07, -0.02** | **<.001** | **-0.09** | **-0.13, -0.05** | **<.001** |
|  | Sleep duration PGS | 0.03 | 0.001-0.05 | .042 | 0.02 | -0.02-0.06 | .392 |
|  | Eveningness PGS | -0.01 | -0.04, 0.01 | .300 | 0.02 | -0.03, 0.06 | .472 |
|  | Schizophrenia PGS | 0.01 | -0.02, 0.04 | .711 | 0.04 | -0.01, 0.08 | .128 |
|  | Short SD PGS | 0.004 | -0.02, 0.03 | .746 | -0.05 | -0.09, -0.01 | .015 |
|  | Long SD PGS | 0.001 | -0.03, 0.03 | .963 | **-0.07** | **-0.11, -0.03** | **.001** |
| **Poor subjective cognition (memory, focus or learning)** | | | | | | | |
|  | Insomnia PGS | **1.14** | **1.07-1.22** | **<.001** | **1.15** | **1.06-1.24** | **<.001** |
|  | Sleep duration PGS | 0.92 | 0.87-0.98 | .013 | 0.94 | 0.86-1.02 | .118 |
|  | Eveningness PGS | 0.99 | 0.93-1.06 | .806 | 0.99 | 0.91-1.07 | .754 |
|  | Schizophrenia PGS | 1.05 | 0.98-1.13 | .181 | **0.86** | **0.78-0.94** | **.001** |
|  | Short SD PGS | 1.04 | 0.97-1.11 | .248 | **1.14** | **1.05-1.24** | **.002** |
|  | Long SD PGS | 1.01 | 0.95-1.07 | .799 | 1.05 | 0.97-1.13 | .283 |

|  |  | **Schizophrenia** | | | **Affective psychoses** | | |
| --- | --- | --- | --- | --- | --- | --- | --- |
|  |  | **OR/B** | **CI** | **p** | **OR/B** | **CI** | **p** |
| **Paired-associated learning** | | | | | | | |
|  | Insomnia PGS | 0.02 | -0.01, 0.05 | .114 | -0.01 | -0.04, 0.03 | .684 |
|  | Sleep duration PGS | 0.02 | -0.01-0.05 | .204 | 0.03 | -0.01-0.06 | .172 |
|  | Eveningness PGS | -0.01 | -0.04, 0.02 | .436 | -0.01 | -0.04, 0.03 | .699 |
|  | Schizophrenia PGS | 0.05 | 0.02, 0.08 | .005 | **0.09** | **0.05, 0.13** | **<.001** |
|  | Short SD PGS | 0.03 | 0.00, 0.06 | .049 | 0.01 | -0.02, 0.05 | .448 |
|  | Long SD PGS | **0.05** | **0.02, 0.07** | **.003** | **-0.03** | **-0.07, 0.01** | **.106** |
| **Reaction time** | | | | | | | |
|  | Insomnia PGS | -0.02 | -0.05, 0.01 | .227 | -0.02 | -0.05, 0.01 | .253 |
|  | Sleep duration PGS | 0.004 | -0.03-0.04 | .795 | 0.03 | -0.002-0.06 | .066 |
|  | Eveningness PGS | 0.004 | -0.03, 0.04 | .823 | -0.02 | -0.05, 0.01 | .125 |
|  | Schizophrenia PGS | 0.03 | -0.01, 0.07 | .178 | **0.07** | **0.03, 0.10** | **<.001** |
|  | Short SD PGS | -0.01 | -0.04, 0.03 | .694 | -0.02 | -0.05, 0.01 | .252 |
|  | Long SD PGS | 0.001 | -0.03, 0.03 | .959 | **0.004** | **-0.03, 0.03** | **.776** |
| **Involuntary hospitalizations** | | | | | | | |
|  | Insomnia PGS | 0.02 | -0.01, 0.05 | .268 | 0.003 | -0.02, 0.02 | .809 |
|  | Sleep duration PGS | -0.001 | -0.03-0.03 | .966 | 0.02 | 0.003-0.05 | .026 |
|  | Eveningness PGS | -0.01 | -0.04, 0.03 | .700 | 0.002 | -0.02-0.02 | .854 |
|  | Schizophrenia PGS | 0.03 | -0.01, 0.07 | .111 | **0.04** | **0.02-0.07** | **<.001** |
|  | Short SD PGS | 0.02 | -0.01, 0.06 | .154 | 0.003 | -0.02, 0.03 | .801 |
|  | Long SD PGS | **0.05** | **0.02, 0.08** | **.003** | 0.001 | -0.02, 0.02 | .913 |
| **Work status** | | | | | | | |
|  | Insomnia PGS | 0.88 | 0.79-0.98 | .022 | 0.90 | 0.81-1.00 | .045 |
|  | Sleep duration PGS | 1.01 | 0.90-1.12 | .891 | 1.01 | 0.91-1.12 | .867 |
|  | Eveningness PGS | 0.94 | 0.88-1.05 | .279 | 0.93 | 0.84-1.04 | .201 |
|  | Schizophrenia PGS | 0.99 | 0.87-1.12 | .849 | 0.89 | 0.79-1.00 | .057 |
|  | Short SD PGS | 0.92 | 0.83-1.03 | .158 | **0.85** | **0.76-0.95** | **.003** |
|  | Long SD PGS | 0.97 | 0.87-1.08 | .575 | 0.96 | 0.86-1.06 | .384 |
| **Clozapine use** | | | | | | | |
|  | Insomnia PGS | 1.08 | 1.02-1.15 | .007 | 0.97 | 0.84-1.11 | .626 |
|  | Sleep duration PGS | 1.00 | 0.95-1.06 | .952 | 1.09 | 0.95-1.26 | .210 |
|  | Eveningness PGS | 1.05 | 0.99-1.12 | .088 | 0.93 | 0.81-1.08 | .346 |
|  | Schizophrenia PGS | **1.14** | **1.06-1.22** | **<.001** | 1.23 | 1.05-1.45 | .012 |
|  | Short SD PGS | 1.06 | 1.00-1.12 | .065 | 0.95 | 0.82-1.10 | .470 |
|  | Long SD PGS | **1.05** | **0.99-1.12** | **.077** | 0.98 | 0.85-1.12 | .742 |

**Supplementary Table 7.** Association between polygenic risk scores and objective variables in different diagnostic groups. Gender and age were included as covariates.

**Supplementary Table 8.** Results from linear regression analyses of PGSs in the diagnostic groups, compared to people with no psychiatric disorder, in the two subgroups of SUPER patients in Finngen sample, and Finngen sample without SUPER patients.

|  | Schizophrenia PGS | | | Sleep duration PGS | | | Insomnia PGS | | | Eveningness PGS | | | Long sleep duration PGS | | | Short sleep duration PGS | | | |
| --- | --- | --- | --- | --- | --- | --- | --- | --- | --- | --- | --- | --- | --- | --- | --- | --- | --- | --- | --- |
| SUPER patients in Finngen sample | OR | CI | p | OR | CI | p | OR | CI | p | OR | CI | p | OR | CI | p | OR | CI | p |  |
| Schizophrenia (N=4708) | 2.54 | 2.46-2.63 | <2×10^-16^ | 1.09 | 1.06-1.13 | 2×10^-9^ | 0.99 | 0.96-1.02 | 0.592 | 1.07 | 1.04-1.10 | 5×10^-6^ | 1.09 | 1.06-1.12 | 3×10^-8^ | 0.96 | 0.93-0.99 | 0.007 |  |
| Affective psychotic disorders (N=3640) | 2.01 | 1.94-2.09 | <2×10^-16^ | 1.06 | 1.03-1.10 | 5×10^-4^ | 1.05 | 1.01-1.08 | 0.009 | 1.03 | 1.00-1.07 | .006 | 1.14 | 1.10-1.18 | 6×10^-14^ | 0.99 | 0.96-1.02 | 0.541 |  |
|  | Schizophrenia PGS | | | Sleep duration PGS | | | Insomnia PGS | | | Eveningness PGS | | | Long sleep duration PGS | | | Short sleep duration PGS | | | |
| Finngen sample without SUPER patients | OR | CI | p | OR | CI | p | OR | CI | p | OR | CI | p | OR | CI | p | OR | CI | p |  |
| Schizophrenia (N=1572) | 2.02 | 1.91-2.14 | <2×10^-16^ | 1.06 | 1.01-1.11 | .023 | 1.01 | 0.96-1.06 | 0.830 | 1.05 | 1.00-1.11 | .005 | 1.11 | 1.05-1.16 | 1×10^-4^ | 0.96 | 0.91-1.01 | 0.092 |  |
| Affective psychotic and bipolar disorders (N=5923) | 1.45 | 1.41-1.49 | <2×10^-16^ | 1.00 | 0.97-1.03 | .982 | 1.09 | 1.06-1.12 | 2×10^-11^ | 1.00 | 0.97-1.02 | .763 | 1.10 | 1.07-1.13 | 2×10^-14^ | 1.08 | 1.06-1.11 | 2×10^-9^ |  |

**Supplementary Figure 1.** STROBE Flowchart of SUPER study sample used in this study.

**SUPER Research project (10411 patients with psychosis)**

Final sample, N=8232

Age unknown, N=8

Over 80 years of age, N= 34

No outcome data, N=159

Patients 18-80 years with known gender and diagnosis, N=8786

Schizophrenia, schizoaffective disorder, bipolar disorder, psychotic depression, N= 8828

Patients with psychosis, N=10411

Unknown diagnosis, N= 538

Other psychosis, N=1045

No genotypic information, N=402
